# Molecular dynamics study of tridymite

**DOI:** 10.1107/S2052252518004803

**Published:** 2018-04-17

**Authors:** Akira Takada, Kathryn J. Glaser, Robert G. Bell, C. Richard A. Catlow

**Affiliations:** a Innovative Technology Research Center, Asahi Glass Company, 1150 Hazawa-cho, Kanagawa-ku, Yokohama, 221-8755, Japan; b Department of Earth Sciences, University College London, Gower Street, London WC1E 6BT, England; c Department of Chemistry, University College London, 20 Gordon Street, London WC1H OAJ, England

**Keywords:** silica, tridymite, molecular dynamics, structure, phase transitions, polymorphs

## Abstract

Structural changes in tridymite have been investigated by molecular dynamics simulation. Two thermal processes were carried out, one cooling from the high-temperature hexagonal structure of tridymite (HP-tridymite) and the other heating from the low-temperature monoclinic structure of tridymite (MX1-tridymite). The former process showed that HP, LHP (low-temperature hexagonal structure), OC (orthorhombic structure with *C*222_1_ symmetry) and OP (orthorhombic structure with *P*2_1_2_1_2_1_ symmetry)-like structures appeared in sequence. In contrast, the latter process showed that MX1, OP, OC, LHP and HP-like structures appeared in sequence. Detailed analysis of the calculated structures showed that the configuration underwent stepwise changes associated with several characteristic modes. First, the structure of HP-tridymite determined from diffraction experiments was identified as a time-averaged structure in a similar manner to β-cristobalite, thus indicating the important role of floppy modes of oxygen atoms at high temperature – one of the common features observed in silica crystals and glass. Secondly, the main structural changes were ascribed to a combination of distortion of the six-membered rings in the layers and misalignment between layers. We suggest that the slowing down of floppy oxygen movement invokes the multistage emergence of structures with lower symmetry on cooling. This study therefore not only reproduces the sequence of the main polymorphic transitions in tridymite, except for the appearance of the monoclinic phase, but also explains the microscopic dynamic structural changes in detail.

## Abstract 2

Polymorphic structural changes in tridymite have been investigated by molecular dynamics simulation. The main polymorphic transitions were reproduced and their mechanisms are explained.

## 1. Introduction   

Tridymite has been long recognized as one of the polymorphs of silica, of which the stable zone is defined clearly between 1140 and 1743 K at ambient pressure on the phase diagram of silica (Heaney, 1994 ▸). Nevertheless, it is not easy to synthesize single crystals of tridymite experimentally without any flux. In addition, even if crystals are obtained, a further complication lies in the existence of different polymorphic and polytypic structures of tridymite, and hence it is not easy to understand the whole family of tridymite from a structural perspective (Heaney, 1994 ▸; Wennemer & Thompson, 1984 ▸; Kihara, 1977 ▸, 1978 ▸; Cellai *et al.*, 1994 ▸; Dollase, 1967 ▸; Dollase & Baur, 1976 ▸; Graetsch & Topalović-Dierdorf, 1996 ▸). Typically, tridymite changes its crystal symmetry from hexagonal through orthorhombic to monoclinic as the temperature decreases. However, even within the same crystal system, several variant structures are produced, with hysteresis depending on the thermal history. Historically, a great deal of experimental effort has been expended to determine all the polymorphic structures, but even now not all are clearly understood.

Additionally, many atomistic simulation studies have been performed on both crystalline and amorphous phases of silica. Nevertheless, to our knowledge there have been no all-atom studies published on tridymite. There has been one theoretical study (Pryde & Dove, 1998 ▸) using the RUM (rigid-unit model), which investigated the main polymorphic structures of tridymite. The analysis based on the RUM proposed the sequence of possible polymorphic transitions qualitatively in terms of low-frequency soft modes. To perform all-atom level simulations, several difficulties present themselves. The first originates from the fact that there are many polymorphic and polytypic structures of tridymite in which the Si—O—Si bond angles were identified to be 180°, a highly atypical geometry in silica. This observation suggests that the calculated structures should be analysed with caution using several structural order parameters. The other difficulty is that a large number of atoms (at least several hundred) are required in the simulation box in order to reproduce the complex structural changes occurring in the polymorphic transitions. Therefore, it is a reasonable solution to use a classical model with interatomic potentials rather than the first-principles method. Accordingly, the choice of potential model for molecular dynamics (MD) simulation also becomes important.

**Figure 1:**
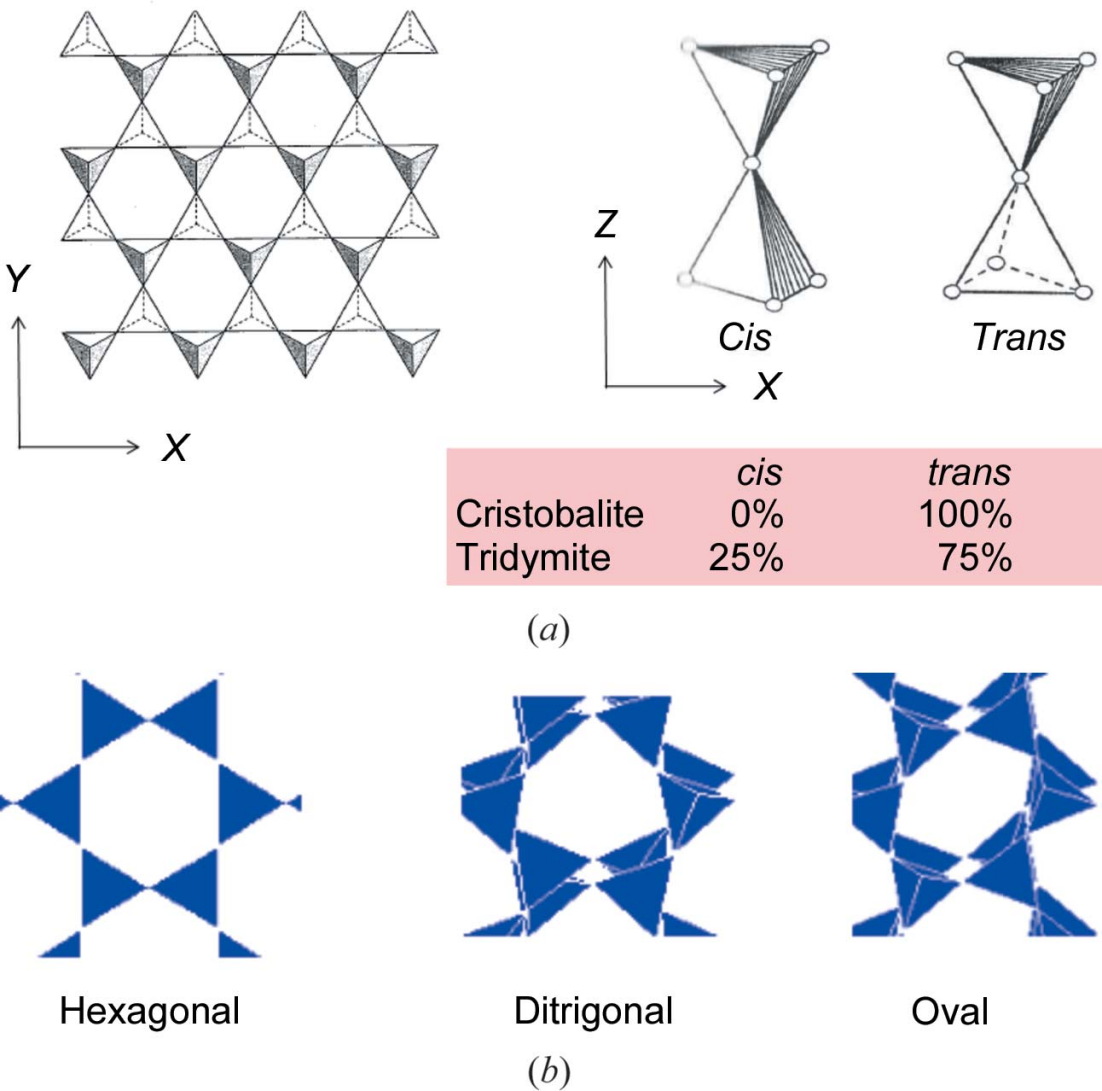
Structural motifs appearing in cristobalite and tridymite.

The structures of tridymite are briefly outlined as follows (Figs. 1 ▸ and 2 ▸). The basic structural unit is a network of SiO_4_ tetrahedra comprised of only six-membered Si—O rings, in an analogous way to cristobalite. It is often stated that two abundant materials, *i.e.* silica and ice, are isostructural: that is to say, tridymite with hexagonal symmetry and cristobalite with cubic symmetry are isostructural with normal ice with hexagonal symmetry and super-quenched ice with cubic symmetry, respectively. Consequently, the stacking of successive layers of tetrahedra differs in the two polymorphs: hexagonal tridymite has the *ABAB* sequence of six-membered rings (Heaney, 1994 ▸; Kihara, 1978 ▸) while cubic cristobalite has the *ABCABC* sequence (Heaney, 1994 ▸). It can be readily speculated that tridymite has many polytypic structures arising from the combination of stacking faults, in a similar manner to the SiC system. In addition, it is not only the crystal symmetry, either hexagonal or cubic, which differs in the two polymorphs, but also the way in which adjacent SiO_4_ tetrahedra are linked. There are two possible configurations of pairs of tetrahedra (Fig. 1 ▸). In one, referred to as ‘*cis*’ or ‘eclipsed’, the oxygen atoms of neighbouring tetrahedra, except the linking oxygen, face each other. In the other, pairs of oxygen atoms are rotated by 180° about the common triad axis with respect to each other, referred to as ‘*trans*’ or ‘staggered’. Cubic cristobalite comprises only the *trans* configuration. In contrast, hexagonal tridymite contains 25% of *cis* and 75% of *trans*.

The above-mentioned hexagonal tridymite (HP-tridymite) is the highest-temperature polymorph. HP-tridymite is transformed to the other polymorphic structures in sequence as the temperature decreases. The polymorphs are categorized by two types of nomenclature: crystal system (hexagonal, orthorhombic, monoclinic) and lattice type (primitive, *C*-centred, and others such as *X*). The major structures are thus named HP (Kihara, 1978 ▸), LHP (low-temperature hexagonal structure) (Cellai *et al.*, 1994 ▸), OC (orthorhombic structure with *C*222_1_ symmetry) (Dollase, 1967 ▸), OP (ortho­rhombic structure with *P*2_1_2_1_2_1_ symmetry) (Kihara, 1977 ▸), MC (monoclinic structure with *Cc* symmetry) (Dollase & Baur, 1976 ▸), and MX-1 (monoclinic superstructure of modulated tridymite with *C*1 symmetry) (Graetsch & Topalović-Dierdorf, 1996 ▸). The sequence of crystal systems appearing from high to low temperature follows this order: hexagonal (HP and LHP), orthorhombic (OC and OP) and monoclinic (MC and MX1). Typically, the polymorph with the higher symmetry is transformed to that with the lower symmetry as the temperature is lowered. The transformation between polymorphs is thought not to be reconstructive but displacive, without any bond breaking.

In this study, MD simulations were used to study the structural transformations of tridymite. Two thermal processes were carried out, one cooling down from the high-temperature hexagonal structure of tridymite (HP-tridymite) and the other heating up from the low-temperature monoclinic structure of tridymite (MX1-tridymite). In both cases, the structural features in HP-tridymite were analysed microscopically at each temperature. In addition, the dominant mechanism of the structural changes in tridymite is discussed.

Finally, we note that, even for structurally simple quartz and cristobalite phases, structural aspects still continue to be a matter of debate. The present authors expect that this first full atomistic simulation of tridymite will encourage further simulation studies of tridymite and other members of the silica crystal family.

## 2. Computational model   

### 2.1. Computational model   

When the classical MD model is used, the choice of interatomic potential model is crucial. Two potential models were initially considered, because they have been applied to MD studies of silica polymorphs more frequently than the other models. One is the ‘BKS’ potential (van Beest *et al.*, 1990 ▸) and the other is the ‘soft’ model (Takada *et al.*, 2004 ▸). These two sets of pair-potential models, which can be easily implemented in any MD code, were critically examined by Soules *et al.* (2011 ▸) and their features compared from an objective point of view. Both potential models include Coulomb interactions; however, there are two major differences between the BKS and soft potentials. The first is that the value of the Coulomb charge on the oxygen atoms is −1.2 for the BKS and −0.65 for the soft model. The second is that the potential form is Buckingham-type for the former and Morse-type for the latter. It is known that the BKS potential overestimates the glass transition temperature by more than 1000 K. On the hand, the soft potential can reproduce it within an error of 100 K due to its small charge value. In addition, a preliminary study performed with the BKS model showed little structural change between 0 and 2000 K, although in the experiment all the phase transitions occur in this temperature range. Moreover, the use of Morse-type potentials does not require any short-range corrections to avoid the spurious attraction forces that occur in the case of the BKS. In addition, the present authors know well the performance of the soft potential model when applied to the silica family. Therefore, only the soft potential model was employed in this study. The potential parameters are shown in Table 1 ▸ (Takada *et al.*, 2004 ▸). A comparative study using different potential models will form the basis of a future study.

The choice of the initial structure of tridymite and the other initial conditions for the MD simulations involved, as noted above, two thermal processes, one cooling down from HP-tridymite and the other heating up from MX1-tridymite. The initial HP-tridymite structure was set up to be isostructural with ice-Ih. The cell parameters are listed in Table 2 ▸. In contrast, the structure of Graetsch & Topalović-Dierdorf (1996 ▸) was used for the initial MX1-tridymite structure in this study. The cell parameters are listed in Table 2 ▸ and internal coordinates were obtained from the Inorganic Crystal Structure Database (ICSD, http://www2.fiz-karlsruhe.de/icsd_home.html; card No. 81382). Several different monoclinic structures such as MC have been proposed experimentally (Heaney, 1994 ▸). Among them, least-common multiples of cell sizes can be easily estimated between the MX1 structure and HP, LHP, OC and OP. The adjusted cell parameters for HP, LHP, OC, OP and MX1 are listed in Table 2 ▸. A comparative study using different experimental MC structures is also a topic for future study. The initial temperature of 1500 K was chosen for the cooling of HP, because this value is close to the experimental glass transition temperature and well above the highest polymorphic transition (between LHP and HP) temperature of 750 K. Although it seems strange that all the Si—O—Si bond angles are 180° in the experimental structure of HP, it is expected that the Si—O—Si bond angles will move to optimal values at 1500 K without bond breaking.

In all simulations, the number of atoms was 864 (288 Si and 576 O) and the initial simulation box was a rectangular parallelepiped. The configuration of HP or MX1 was used. The initial configuration of HP was converted from hexagonal to orthorhombic. The lattice parameters are *a* = 30.30 Å, *b* = 26.24 Å, *c* = 16.54 Å and α = β = γ = 90.00°. The initial configuration of MX1 was monoclinic. The lattice parameters are *a* = 30.60 Å, *b* = 25.80 Å, *c* = 16.43 Å, α = γ = 90.00° and β = 91.54°. The number of atoms in the unit cell differs among the polymorphic structures; however, the number of atoms in the simulation box (*i.e.* 864) was chosen so that it becomes an integer multiple of all the numbers of atoms among the polymorphic structures. During the simulation, the shape and volume of the simulation box were allowed to change within the NPT ensemble. We used the *DL_POLY* code, a general-purpose MD simulation package developed at the Daresbury Laboratory, UK (Forester & Smith, 2001 ▸). The primary advantage of this code is that a variety of potential forms, thermostats and barostats are available. To control temperature and pressure, the Nosé–Hoover thermostat and the Hoover barostat were used.

The procedures used in the calculations were as follows. The time step was 1 fs. In the case of the cooling of HP, the initial configuration of HP was stabilized at 100 ps at 1500 K. Then the structure was cooled down to 100 K stepwise in 100 K and 100 ps intervals, and finally the structure was stabilized at 10 K. The structure was stabilized for 10 ps at each temperature. The nominal cooling rate was 1 K ps^−1^ between each change of 100 K. Next, the cooled configuration was reheated up to 1500 K to check the reversibility. At each temperature step, the calculated structure was analysed. In the case of MX1, which is thought to be the lowest-temperature polymorphic structure, the heating cycle was executed first and the cooling cycle was then executed using the same intervals as for HP. In addition, in the case of OC-tridymite and OP-tridymite, whose stable temperature regions observed experimentally are in the middle range (∼380–750 K), there was no guarantee beforehand that each initial experimental structure would be stable in the aforementioned temperature range. Hence, instead of applying the heating or cooling cycle, the experimental configuration was exposed to each temperature instantaneously and stabilized at 10 ps. Only the simulation of MX1 showed some irreversible behaviour between the heating and cooling cycles. To check the effect of the heating and cooling rates, the interval was changed from 100 ps to 1000 ps and the latter results are shown in this paper. However, little difference was observed. In this study, a simulation of MC was omitted, because the atomic arrangements are very different from the other polymorphic structures. After the stabilization process, the properties were calculated averaged over a sampling time of 50 ps so that they converged within an error of a few percent.

### 2.2. Structural order parameters   

The structures are intricately transformed in sequence as the temperature is lowered. The structural transformation occurs in different ways. To analyse such transformations, it is important to choose appropriate structural parameters. In this study, five structural parameters were calculated at each temperature step. The first is the density, as the change in density always correlates with a noticeable change in structure. The second is the Si—O—Si bond angle. The SiO_4_ tetrahedron is quite rigid; however, the connections between SiO_4_ tetrahedra change along with the distortion of the whole network. The general structural motif of the tridymite family comprises *ABAB* stacking. It is important to analyse the angles in plane (in the *XY* layer) and out of plane (in the *Z* direction) separately.

The third parameter is the torsion angle that defines the *cis*- and *trans*-conformations. The difference between the ideal structures of cristobalite and tridymite is characterized by the ratio of the *cis*- to the *trans*-conformation. To analyse these conformations, the O—Si—Si—O torsion angle is calculated. For each dimer comprised of two SiO_4_ tetrahedra, three torsion angles (TAs) are calculated and averaged. In the case of ideal *cis*- and *trans*- conformations, the corresponding angles are 0.0° and 60.0°, respectively.

The fourth parameter is the alignment between two layers (*A* and *B*). To quantify the degree of misalignment between two layers, we consider two sets of centres of mass for two six-membered rings: one lies in layer *A* and the other in layer *B*. The two centres of mass were next projected onto the *XY* plane and the distance between the two projected points was calculated. In the case of non-misalignment, the distance is zero. Therefore, the difference is defined as the length of misalignment of the layers.

The fifth parameter is the emergence of ditrigonal and oval six-membered rings. In experiment, some of the six-membered rings on the *XY* plane convert from regular hexagonal in HP and LHP into ditrigonal (corrugated hexagonal) in OC and then oval (elongated in one direction) in OP and MC, as shown in Fig. 1 ▸.

## 3. Results   

### 3.1. Density and lattice parameters   

The calculated density and lattice parameter profiles of the simulations starting from HP are shown in Figs. 3(*a*) ▸ and 4 ▸(*a*). The calculated lattice parameters *a*, *b* and *c* are given by the ratio of the initial experimental HP values. In the case of the HP profile, the lattice angles are not shown, because the changes in lattice angle were less than 0.01° across the whole temperature profile. The measured densities vary, depending on the crystal system and observed temperature, between 2.18 gm^−3^ in HP and 2.26 g cm^−3^ in MX1. Compared with the experimental values, the calculated densities fluctuate more, but the variation always stays within 10%. The cooling and heating cycles show almost reversible behaviour. The profile bends and only a small hysteresis is observed around 1000 K, suggesting that the phase transition occurs. As for the lattice parameters, all three change differently below 1000 K, with the largest change in the *a* parameter. Next, the calculated profiles of the simulations starting from MX1 are shown in Figs 3 ▸(*b*), 4 ▸(*b*) and 4 ▸(*c*). The calculated cell parameters and densities at representative temperatures are tabulated in Tables 2 ▸ and 3 ▸, respectively. During the heating cycle, three turning points are observed at ∼400–500, ∼700–800 and ∼900–1000 K in the density profiles. The heating profile of MX1 merges with the cooling profile of HP above 1000 K. The corresponding turning points are also recognized in both lattice parameter and angle profiles. In contrast with the HP profile, strong hysteresis is observed for the MX1 profile in the lower-temperature region between the heating and cooling profiles. In particular, the change in lattice parameter *a* is prominent. The cooling profile of MX1 is almost the same as that of HP-tridymite. Thirdly, the calculated density profiles of LHP, OC and OP are shown in Fig. 3 ▸(*c*). It is interesting to note that these three density profiles are almost the same as that calculated for HP-tridymite. In other words, the structures starting from LHP, OC and OP would be almost the same as those of HP at the corresponding temperature. The other important feature is that the experimental initial monoclinic cell converts to orthorhombic even at 10 K, although the other features suggest that the local structure still keeps the MC-like motif, as discussed below.

### 3.2. Si—O—Si bond angles   

The distributions of the Si—O—Si bond angles calculated based on the time-averaged configuration in HP are shown in Fig. 5 ▸. It is important to note that the single angle calculated based on the time average of three coordinates (Si, O and Si) differs from the simple average over time of all Si—O—Si angles. At 1500 K, almost all the Si—O—Si bond angles are 180°, which means the configuration corresponds to that of ideal HP. When the temperature decreases down to 900 K, the distribution of Si—O—Si bonds that are in plane (*XY* layer) splits into two peaks. Such peak splitting corresponds with the in-plane distortion of Si—O six-membered rings observed experimentally in LHP. Next, the lowest peak at around 150° is further split into two peaks at 700 K and this pattern persists down to 10 K. Such splitting around 150° is a characteristic found in OC, OP, MC or MX1. On the other hand, no splitting is observed for the out-of-plane bonds as the temperature decreases.

The value of the calculated bond angles based on the time-averaged configuration is suitable for comparison with diffraction data. However, the values of the bond angles at each instant are quite different. The distribution of Si—O—Si bond angles calculated based on the snapshot at 1500 K is shown in Fig. 6 ▸. It is clear that the instantaneous Si—O—Si bond angle is not 180° even though the diffraction studies suggested such an angle.

The distributions of Si—O—Si bond angles calculated based on the time-averaged configuration in MX1 are shown in Fig. 7 ▸. The distribution in the starting configuration at 10 K in Fig. 7 ▸ appears to be different from that cooled from HP and shown in Fig. 5 ▸(*a*). When the temperature increases up to 3700 K, the in-plane (*XY*) distribution is split into two, as shown in Fig. 7 ▸. At 900 K, the lower two peaks shift to the larger values. Finally, at 1500 K the distribution becomes very similar to that of ideal HP. In summary, the in-plane single-peak distribution of Si—O—Si bond angles persists below 700 K, in contrast with the case of HP cooling, suggesting that the structure below 700 K differs between MX1 heating and HP cooling.

### 3.3. *Cis*–*trans* conformation   

The distribution of the O—Si—Si—O torsion angles during the cooling cycle of HP is shown in Fig. 8 ▸. At 1500 K, all the torsion angles in plane are 60°, which signifies the *trans*-conformation. In contrast, all the angles out of plane are 0°, *i.e.* in the *cis*-conformation. When the temperature decreases to 900 K, the in-plane angles split into two peaks, although no change in the out-of-plane angles is observed. At 10 K, the in-plane angles are split into three peaks. The in-plane angles suggest that the *trans*-conformation deviates from the ideal value of 60°. In contrast, the out-of-plane angles suggest that there is only a small deviation from the ideal *cis* value of 0°.

Next, the distribution of O—Si—Si—O torsion angles during the heating of MX1 is shown in Fig. 9 ▸. The starting distribution at 10 K has more peaks and the shape is more distorted than that of HP. The out-of-plane peak deviates from the ideal value of 0°. As the temperature increases up to 300 K, the number of peaks for the in-plane angles reduces to two. At 700 K, the out-of-plane peak moves to the ideal value of 0°. Finally, above 1100 K, the distribution reverts to the same as that of HP.

### 3.4. Misalignment between layers   

The profile of misalignment is shown in Fig. 10 ▸. The mis­alignment is calculated during the cooling process of HP and the heating process of MX1. In both cases, the misalignment occurs below 900 K and the magnitude increases as the temperature is lowered.

### 3.5. Shape of the six-membered rings   

**Figure 2:**
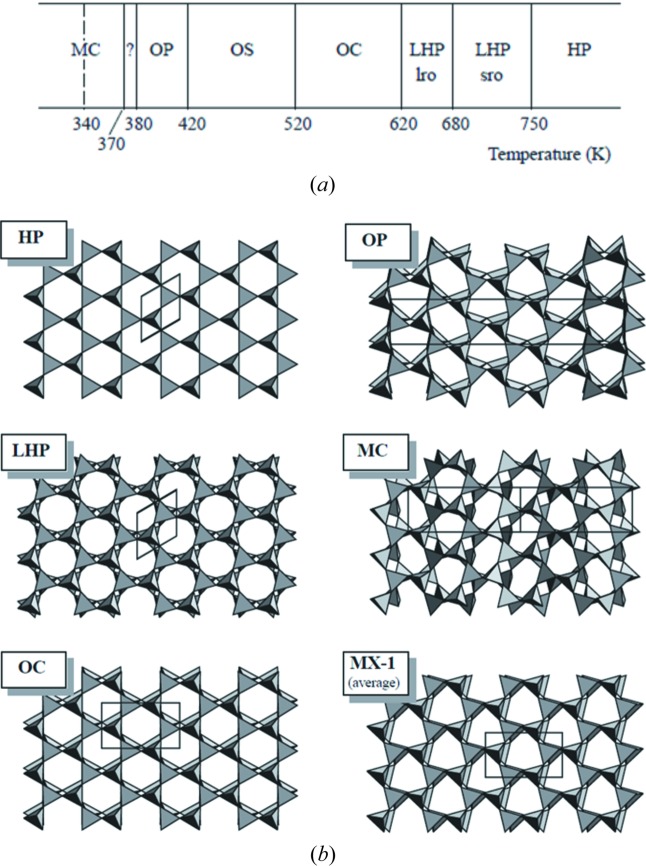
Sequence of tridymite phases and crystal structures. Reproduced with permission from Pryde & Dove (1998 ▸), copyright (1998) Springer Nature.

**Figure 3:**
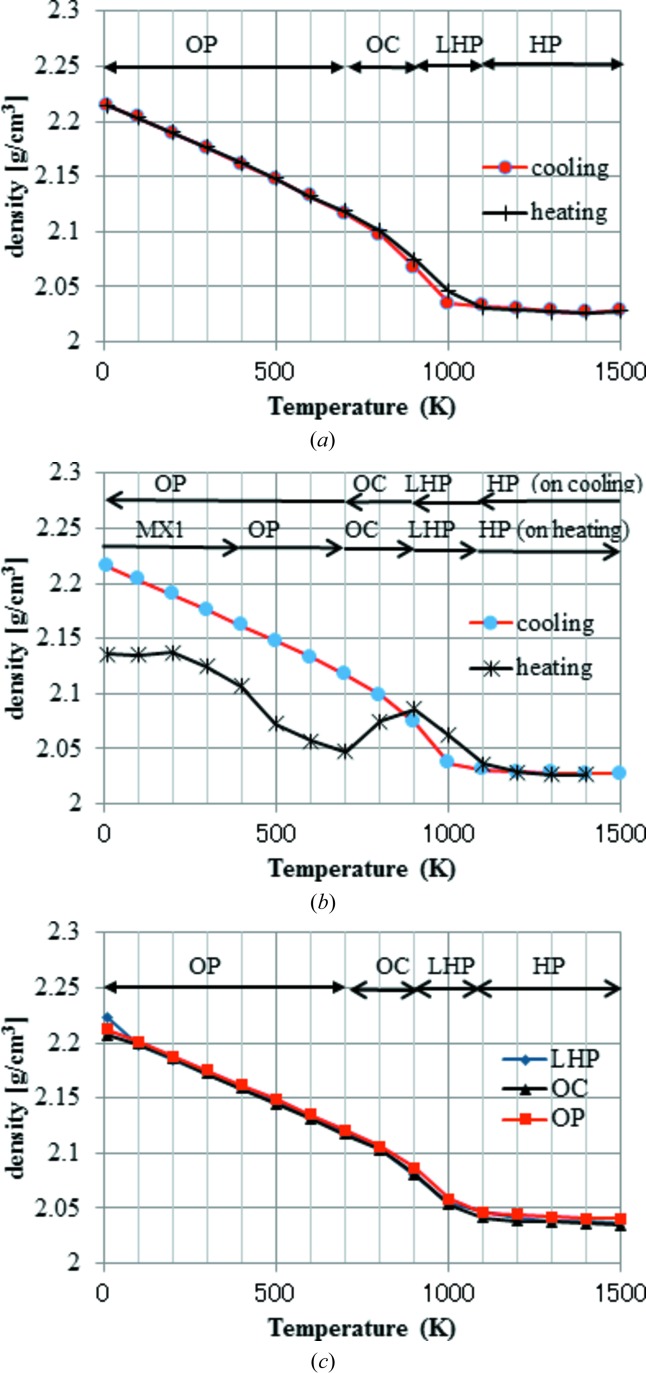
Calculated profiles of density. (*a*) Starting from the HP-tridymite structure. (*b*) Starting from the MX1-tridymite structure. (*c*) Starting from the LHP-, OC- or OP-tridymite structure. Each profile corresponds to that during either heating or cooling, with the exception of those in panel (*c*), which correspond to heating only. Conjectured structural types appearing during the simulation are indicated, accompanied by arrows.

**Figure 5:**
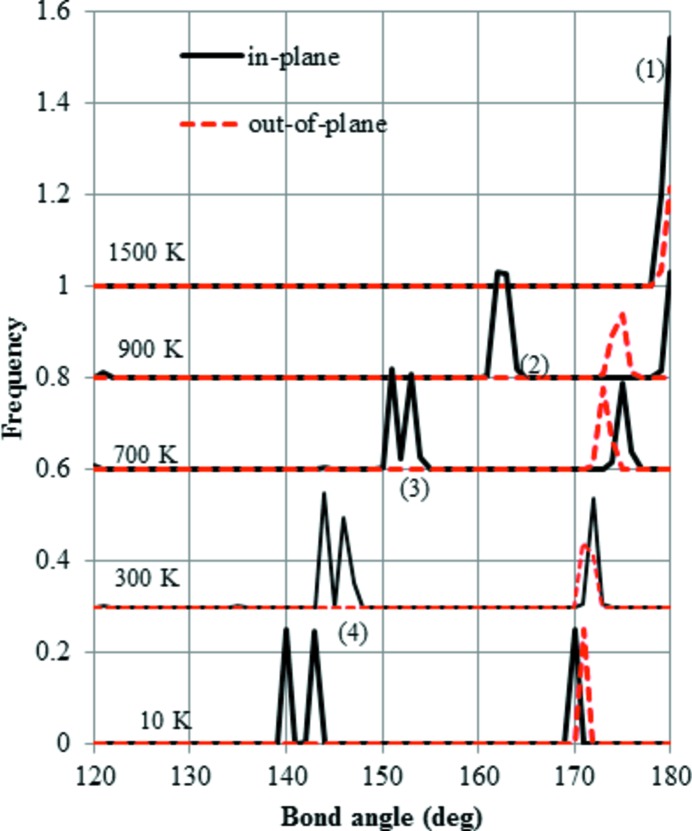
The distribution of Si—O—Si bond angles during cooling, starting from the HP-tridymite structure. Two distributions were calculated, *i.e.* that in the *XY* plane and that out of the *XY* plane, each based on its average coordinates at temperatures of 1500, 900, 700 and 10 K. (1) Peak characteristic of the HP structure. (2) Peak characteristic of LHP. (3) Peak characteristic of OP. (4) Lower shift of the peak characteristic of OP.

**Figure 6:**
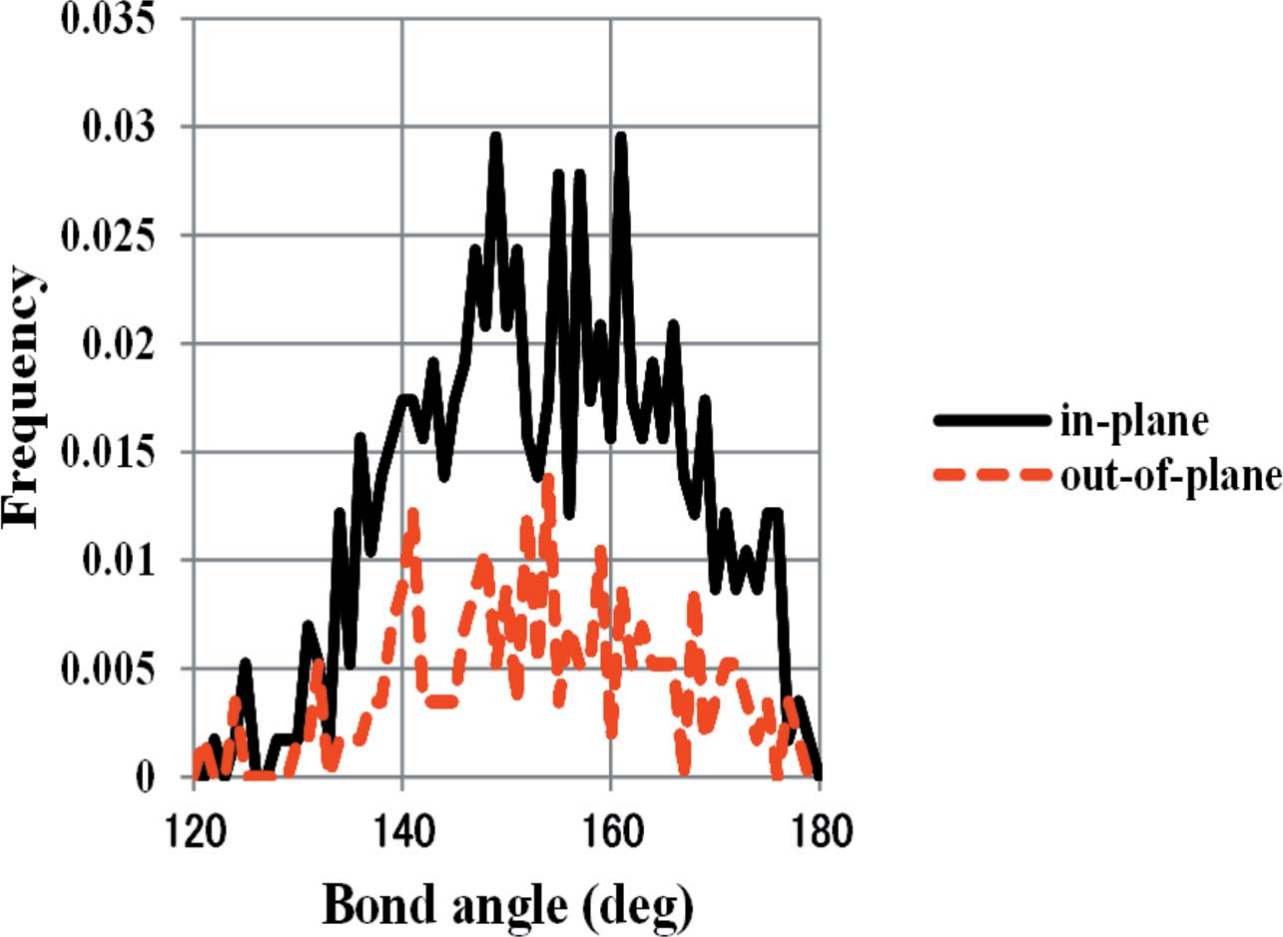
Distribution of Si—O—Si bond angles sampled instantaneously at 1500 K for HP-tridymite.

The calculated projections of the *XY* and *YZ* planes at representative temperatures are shown in Fig. 11 ▸. Only the heating process from the MX1 structure is analysed. Up to 300 K, the structure keeps an MX1-like structure motif, because all the shapes of the six-membered rings on the *XY* plane are ditrigonal, as shown in Fig.11 ▸(*a*). This is in agreement with experiment (Fig. 2 ▸). When the structure is heated up to 500 K, all six-membered rings in the *XY* plane become hexagonal-like ditrigonal, as shown in Fig. 11 ▸(*c*). However, experiment suggests that the OP structure is two thirds ditrigonal and one third oval (Fig. 2 ▸) and the oval part of the structure is not reproduced. Further heating up to 800 K converts the structure into oval, as shown in Fig. 11 ▸(*e*). In contrast, experiment suggests that the OC structure is only ditrigonal (Fig. 2 ▸). Finally, at 900 K, all the shapes become hexagonal, in agreement with experiment (Figs. 11 ▸ *g*) and 11 ▸ *i*). To evaluate the deviation from a regular hexagon, an appropriate index is thought to be the Si—O—Si angle. The angle is 180° for HP or about 163° for LHP, as shown in Fig. 5 ▸.

In the reverse process of cooling, the profiles of the shape distributions above 700 K are the same as with heating. However, even below 700 K, all the oval shapes are kept down to 10 K. Here, only the cross section of *XY* is shown in Fig. 11 ▸(*k*). This result supports our conclusion that the structure below 700 K differs between MX1 heating and cooling, the latter being almost the same as with HP cooling.

## 4. Discussion   

As noted in Section 3.1, the structural change starting from HP at high temperature is almost reversible. In contrast, the change from MX1 is irreversible. One possible interpretation is that the calculated structure cooled from HP fell into the OP-like structure and did not reach the monoclinic family of MC or MX1. In other words, this study suggests the tree diagram of structural change has two lineages; one is HP, LHP, OC and OP, and the other is MX1 and possibly MC which was not simulated in this paper. This result corresponds with the conclusions obtained from the RUM analysis (Pryde & Dove, 1998 ▸). To check the energy stability, energy minimization calculations at 0 K were applied to the OC, OP and MX1 experimental structures. The calculated energies are −23.157 eV for OC, −23.170 eV for OP and −23.156 eV for MX1. Therefore, MX1 is the most unstable compared with OC, OP and MC. This is the reason why the MX1 structure was not obtained during the cooling process.

Firstly, the structural change cooling down from HP is considered. The starting structure at 1500 K is the same as the ideal HP (Kihara, 1978 ▸). It is interesting to note that the bond angles of Si—O—Si measured instantaneously are not 180°. However, the experimental value of 180° can be reproduced using the time-averaged atomic coordinates. The importance of dynamic structures has been stressed in earlier papers (Takada *et al.*, 2007 ▸, 2008 ▸). These structural features are common to β-cristobalite in which the Si—O—Si bond angles are also observed to be 180°. When the structure is cooled down to 900 K, the peak of the in-plane bond angle of Si—O—Si splits into two, indicating large changes only in the in-plane configuration. These results agree with the HP–LHP transition. The temperature of 900 K is close to the experimental transition temperature of 750 K (Cellai *et al.*, 1994 ▸). It is interesting to note that only one temperature-induced structural change, denoted the α–β transition, is known in the case of cristobalite. We can speculate that the structural change in cristobalite occurs all at once at a single temperature point, because the stacking in the structure is isotropic and there is no preference in the direction of structural change. In contrast, the structure of HP-tridymite has the anisotropic stacking sequence *ABAB*. The orientation of paired tetrahedra is *trans* or *cis* depending on whether it is in plane or out of plane. Chemical intuition suggests that the *trans* orientation is more stable than the *cis*, because the repulsion between basal oxygen atoms is reduced. Consequently, the active movements of in-plane oxygen atoms are reduced at higher temperature compared with those out of plane. To summarize, the HP–LHP transition involves a change in the motions of in-plane oxygen atoms. Although the internal configuration changes gradually, there is little change in the lattice parameters.

Next, down to 800 K the misalignment of layers begins as shown in Fig. 11 ▸. Such misalignment is not observed experimentally in HP or LHP, but it is in OC (Dollase, 1967 ▸). The calculated results suggest that the LHP–OC transition occurs at around 800 K. The experimental transition temperature is around 620 K (Dollase, 1967 ▸). The experimental and calculated values of the misalignment in OC are 0.56 and 0.59 Å, respectively. Immediately after the HP–LHP transition, in which splitting of the peak of the in-plane bond angle into two occurs, the peak of the out-of-plane angle shifts towards a lower value of around 175° and the value gradually reduces. We can speculate that some of the out-of-plane oxygen atoms reduce their motions and the tetrahedral sheets are offset so that the structure cannot hold up against the repulsion between basal oxygen atoms. The OC–OP transition is not clearly recognized. In experiment, all the Si—O—Si bond angles are above 160° for OC (Dollase, 1967 ▸). In contrast, the bond angle is distributed between 145 and 176° for OP (Kihara, 1977 ▸). In our simulations, the distribution is only above 160° at 900 K; however, the lower peaks shift down to around 150° at ∼700 K. This observation suggests that the change from an OC- to an OP-like structure occurs gradually not abruptly. The other important feature is that the lower peak of the in-plane bond angles splits into two below ∼700 K, which suggests the in-plane six-membered rings are more distorted from the regular shape of a hexagon. Finally, at 10 K the calculations suggest that the OP-like structure does not transform into the MC (Dollase & Baur, 1976 ▸) or MX1 (Graetsch & Topalović-Dierdorf, 1996 ▸) structure due to energetic factors. Differential scanning calorimetry (DSC) results showed two first-order anomalies in the specific heat at around 380 and 370 K (Cellai *et al.*, 1994 ▸), which suggests some recognizable change in the structure should appear at those temperatures. However, the slowing down of oxygen atoms is gradual in our calculations and no clear structural transition is found.

**Figure 7:**
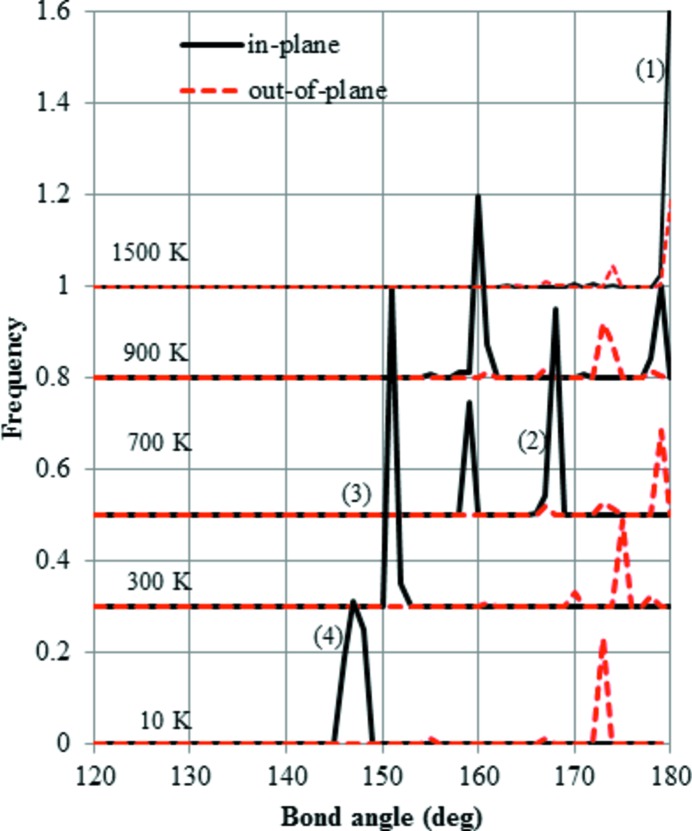
Distribution of Si—O—Si bond angles during heating, starting from the MX1-tridymite structure. Two distributions were calculated, *i.e.* that in the *XY* plane and that out of *XY* plane, each based on its average coordinates at temperatures of 10, 300, 700 and 1100 K. (1) Peak characteristic of the HP structure. (2) Peak characteristic of OC. (3) Peak characteristic of OP. (4) Peak characteristic of MX1.

**Figure 8:**
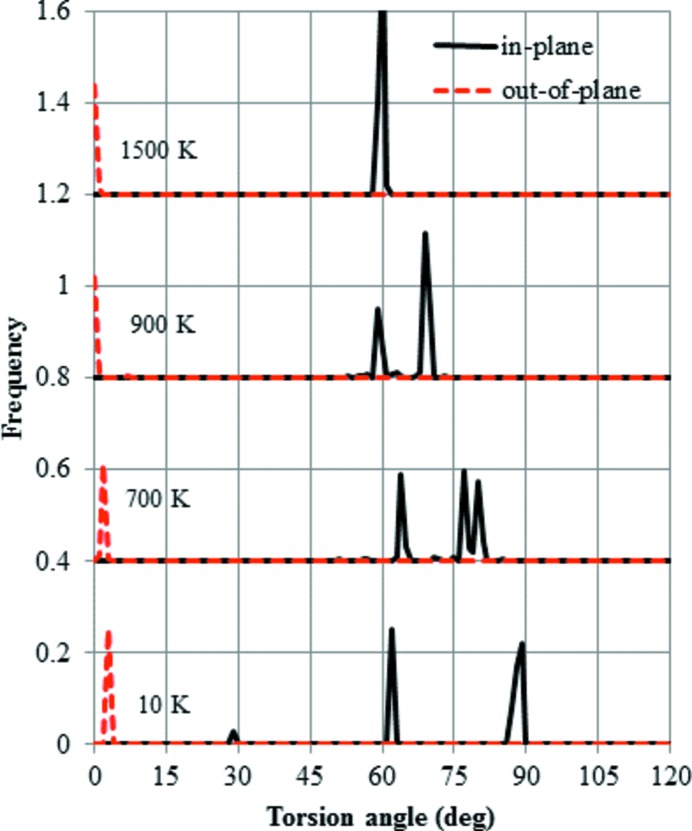
Distribution of O—Si—Si—O torsion angles during cooling, starting from the HP-tridymite structure. Two distributions were calculated, *i.e.* that in the *XY* plane and that out of the *XY* plane, each based on its average coordinates at temperatures of 1500, 900, 700 and 10 K.

**Figure 9:**
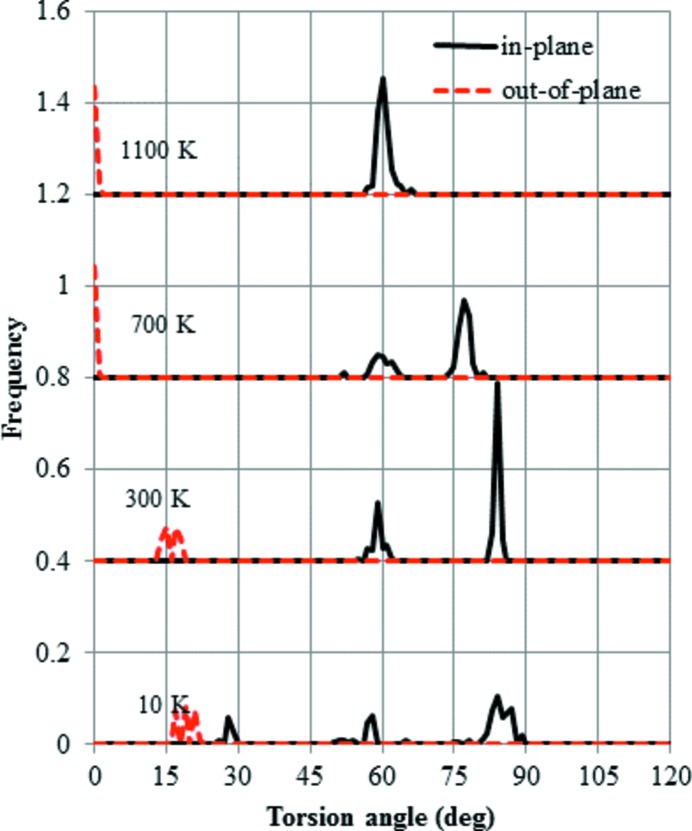
Distribution of O—Si—Si—O torsion angles during heating, starting from the MX1-tridymite structure. Two distributions were calculated, *i.e.* that in the *XY* plane and that out of the *XY* plane, each based on its average coordinates at temperatures of 10, 300, 700 and 1100 K.

**Figure 10:**
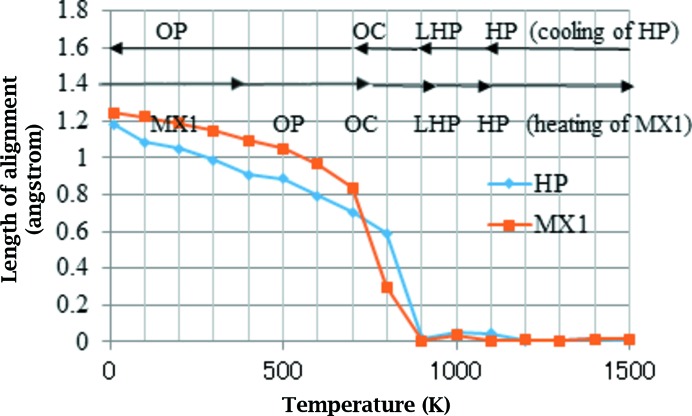
Misalignment between layers. Each profile corresponds to that during either heating or cooling. Conjectured structural types appearing during the simulation are indicated, accompanied by arrows

**Figure 11:**
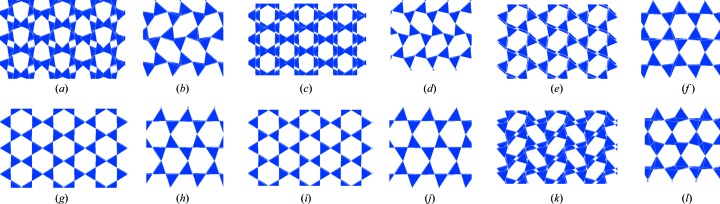
Projection views of calculated structures. (*a*) *XY* cross section at 300 K during heating from the MX1 structure. (*b*) *YZ* cross section at 300 K during heating from the MX1 structure. (*c*) *XY* cross section at 500 K during heating from the MX1 structure. (*d*) *YZ* cross section at 500 K during heating from the MX1 structure. (*e*) *XY* cross section at 800 K during heating from the MX1 structure. (*f*) *YZ* cross section at 800 K during heating from the MX1 structure. (*g*) *XY* cross section at 900 K during heating from the MX1 structure. (*h*) *YZ* cross section at 900 K during heating from the MX1 structure. (*i*) *XY* cross section at 1500 K during heating from the MX1 structure. (*j*) *YZ* cross section at 1500 K during heating from the MX1 structure. (*k*) *XY* cross section at 300 K during cooling from the HP structure. (*l*) *YZ* cross section at 300 K during cooling from the HP structure

Next, we discuss the structural changes on heating up from the MX1 structure The many peaks observed at 10 K in both the distribution of Si—O—Si bond angles (Fig. 7 ▸ *a*) and that of torsion angles (Fig. 9 ▸ *a*) indicate that this structure is more distorted than that cooled from HP. When the temperature is raised up to ∼300 K, the lower peaks of Si—O—Si bond angles located at 150° shift to the higher value as shown in Fig. 7 ▸(*b*). The interpretation is that there is a gradual change of structure from MX1 to OP. Further heating up to 700 K (Fig. 7 ▸ *c*) moves the lower peaks to above 160°, suggesting the structure changes from OP to OC. The near disappearance of misalignment at 900 K means that the structure transforms from OC to LHP. Finally, all the Si—O—Si bond angles are now ∼180° and the transition from LHP to HP has occurred. Only the lattice parameter *a* showed a large change between 700 and 800 K during heating. The reason for this behaviour will be the topic of a future study.

In summary, heating up from MX1 reproduces the main sequence of structural changes occurring in tridymite reasonably well; however, cooling the structure down from HP results in it being frozen in the OP-like structure. We may speculate the latter is due to two possible reasons. The first is the insufficiency of the potential model, in which context we note that there are still discrepancies in terms of Si—O—Si bond angle and transition temperature. The second is the structural diversity of tridymite. In the real tridymite system, an abundance of different structures have been observed experimentally around this temperature zone. For example, orthorhombic tridymite exhibits many superstructures and room-temperature terrestrial tridymite shows different pseudo-orthorhombic structures (Heaney, 1994 ▸). There is also a possibility that the calculated structure after cooling down from HP may correspond to one of these pseudo-orthorhombic structures. In the simulation, the calculation of the other monoclinic structures, the effects of superstructure size on stacking faults and the effect of quenching rates on reaching metastable structures will be investigated in a future study.

## 5. Conclusions   

In our MD study of the structure of tridymite, not only was the high-temperature structure of HP reproduced, but also the complex structural changes occurring from MX1 through OP, OC, LHP to HP were explained. However, the model cooled down from HP failed to turn into a monoclinic structure at the final stage, because the structure was trapped and frozen as an OP-like structure.

The structural transformations invoked by the thermal change were shown by several microscopic features. First, the Si—O—Si bond angle of 180° identified by diffraction study originates from the time-averaged positions of oxygen atoms which have floppy movements in a similar manner to β-cristobalite. Secondly, the sequence of structural transformations is a combination of several characteristic features. When the structure of MX1 was heated up to 300 K, the distortion of the in-plane six-membered rings changed such that the Si—O bond-angle peak was split into two. At 700 K, the lower peak of the Si—O bond angle increased from 150° to 160° as the in-plane six-membered rings adopted a more regular shape. However, it still left a misalignment between the layers. When the temperature was raised up to 900 K, the misalignment disappeared and the structure turned out to be LHP. Finally, at 1110 K the remaining distortion of the in-plane six-membered rings disappeared and the most symmetric HP structure was formed. Our study suggests that the main difference in temperature-induced transitions between tridymite and cristobalite originates from the difference in stacking. In other words, the six-membered Si—O rings behave differently in plane and out of plane.

The investigation of structural changes in tridymite has wider relevance, not only to cristobalite and silica glass, but also to other isomorphic systems like ice or SiC. Future more detailed investigation of the two basic structural systems (cubic and hexagonal) common in silica and ice is expected to lead to a deeper understanding of some of the most fundamental questions in structural chemistry.

## Figures and Tables

**Figure 4:**
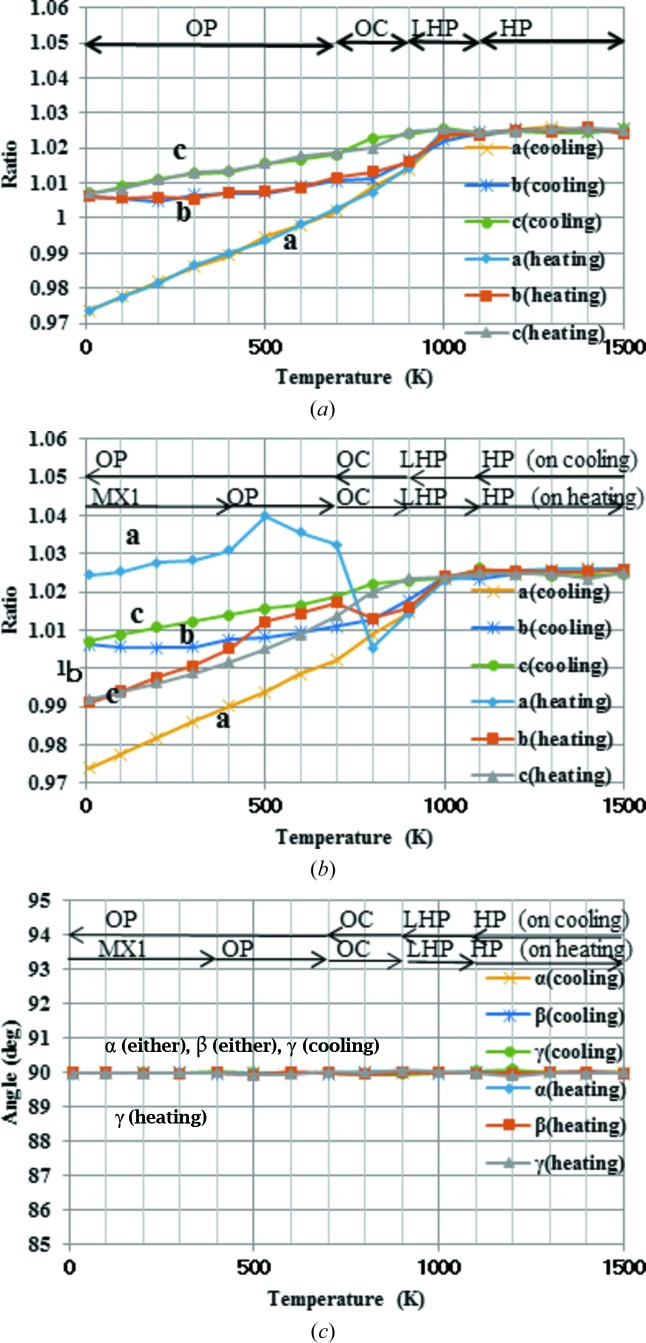
Calculated profiles of lattice parameters. (*a*) Calculated profiles of lattice lengths starting from the HP-tridymite structure. (*b*) Calculated profiles of lattice lengths starting from the MX1-tridymite structure. (*c*) Calculated profiles of lattice angles starting from the MX1-tridymite structure. Each profile corresponds to that during either heating or cooling. Conjectured structural types appearing during the simulation are indicated, accompanied by arrows.

**Table 1:** Parameters for interatomic potentials Charge *q*(Si) = 1.3, *q*(O) = −0.65. The Morse potential *V* (eV) = *A*{1 − exp[−*B*(*r* − *C*)]}^2^ − *A*, where *r* is the atomic radius (Å).

	*A* (eV)	*B* (Å^−1^)	*C* (Å)
Si—O	1.995970	2.65180	1.6280
O—O	0.023272	1.33310	3.7910
Si—Si	0.007695	2.04666	3.7598

**Table 2:** Comparison of the simulated lattice parameters with the experimental values In the simulation, the structure is heated up from experimental MX1-tridymite and cooled down. The upper and lower rows correspond to (*a*, *b*, *c*) and [α, β, γ], respectively. The temperature values for each structure are the same as those in Table 3 ▸. Initial supercell settings of (6,5,2), (6,5,2), (6,3,2), (6,1,2) and (6,3,2) are used for simulations on HP, LHP, OC, OP and MX1, respectively.

	Simulation on heating	Simulation on cooling	Experiment
HP	(31.0, 26.8, 16.9)	(31.0, 26.9, 16.9)	(30.3, 26.2, 16.5)
	[90.0, 90.0, 90.1]	[90.0, 90.0, 90.0]	[90.0, 90.0, 90.0]
LHP	(30.7, 26.7, 16.9)	(30.7, 26.7, 16.9)	(30.3, 26.2, 16.5)
	[90.0, 90.0, 90.0]	[90.0, 90.0, 90.0]	[90.0, 90.0, 90.0]
OC	(31.3, 26.7, 16.8)	(30.4, 26.5, 16.8)	(30.2, 26.2, 16.5)
	[90.0, 90.0, 90.0]	[90.0, 90.0, 90.0]	[90.0, 90.0, 90.0]
OP	(31.4, 26.6, 16.6)	(30.1, 26.4, 16.8)	(29.9, 26.2, 16.4)
	[90.0, 90.0, 90.0]	[90.0, 90.0, 90.0]	[90.0, 90.0, 90.0]
MX1	(31.2, 26.3, 16.5)	(no appearance)	(30.3, 25.8, 16.4)
	[90.0, 90.0, 90.0]		[90.0, 91.5, 90.0]

**Table 3:** Comparison of simulated densities with those obtained experimentally (g cm^−3^) In the simulation, the structure was heated up from experimental MX1-tridymite and cooled down, then the appropriate polymorph name was allocated.

	Simulation on heating	Simulation on cooling	Experiment
HP	2.038	2.038	2.185
	*T* = 1000 K	*T* = 1000 K	*T* = 733 K
LHP	2.075	2.074	2.188
	*T* = 900 K	*T* = 900 K	*T* = 673 K
OC	2.051	2.117	2.199
	*T* = 700 K	*T* = 700 K	*T* = 493 K
OP	2.069	2.148	2.236
	*T* = 500 K	*T* = 500 K	*T* = 428 K
MX1	2.141	(no appearance)	2.257
	*T* = 200 K		*T* = 295 K
